# Attributes That Influence Human Decision-Making in Complex Health Services: Scoping Review

**DOI:** 10.2196/46490

**Published:** 2023-12-20

**Authors:** Nandini Doreswamy, Louise Horstmanshof

**Affiliations:** 1 Faculty of Health Southern Cross University Lismore Australia; 2 National Coalition of Independent Scholars Canberra Australia

**Keywords:** human attributes, human decision-making, rationality, rational decision-making, health policy, health regulation, health services

## Abstract

**Background:**

Humans currently dominate decision-making in both clinical health services and complex health services such as health policy and health regulation. Many assumptions inherent in health service models today are underpinned by Ramsey’s Expected Utility Theory, a prominent theory in the field of economics that is rooted in rationality. Rational, evidence-based metrics currently dominate the culture of decision-making in health policy and regulation. However, as the COVID-19 pandemic has shown, rational metrics alone may not suffice in making better policy and regulatory decisions. There are ethical and moral considerations and other complex factors that cannot be reduced to evidence-based rationality alone. Therefore, this scoping review was undertaken to identify and map the attributes that influence human decision-making in complex health services.

**Objective:**

The objective is to identify and map the attributes that influence human decision-making in complex health services that have been reported in the peer-reviewed literature.

**Methods:**

This scoping review was designed to answer the following research question: what attributes have been reported in the literature that influence human decision-making in complex health services? A clear, reproducible methodology is provided. It is reported in accordance with the PRISMA-ScR (Preferred Reporting Items for Systematic Reviews and Meta-Analyses Extension for Scoping Reviews) standards and a recognized framework. As the topic of interest merited broad review to scope and understand literature from a holistic viewpoint, a scoping review of literature was appropriate here. Inclusion and exclusion criteria were developed, and a database search undertaken within 4 search systems—ProQuest, Scopus, PubMed, and Web of Science.

**Results:**

The results span 46 years, from 1976 to 2022. A total of 167 papers were identified. After removing duplicates, 81 papers remained. Of these, 77 papers were excluded based on the inclusion and exclusion criteria. The remaining 4 papers were found to be relevant. Citation tracking was undertaken, identifying 4 more relevant papers. Thus, a total of 8 papers were included. These papers were reviewed in detail to identify the human attributes mentioned and count the frequency of mentions. A thematic analysis was conducted to identify the themes.

**Conclusions:**

The results highlight key themes that underline the complex and nuanced nature of human decision-making. The results suggest that rationality is entrenched and may influence the lexicon of our thinking about decision-making. The results also highlight the counter narrative of decision-making underpinned by uniquely human attributes. This may have ramifications for decision-making in complex health services today. The review itself takes a rational approach, and the methods used were suited to this.

**International Registered Report Identifier (IRRID):**

RR2-10.2196/42353

## Introduction

### Background

Health care can be broadly divided into clinical health services, health policy, and health regulation. It is important to make a clear distinction among these 3 spheres, to ensure clarity in discussions, arguments, and decisions relating to health care. Clinical health services refer to the diagnosis, treatment, rehabilitation, palliation, and prevention of disease, and they focus, for the most part, on individual health care. Health policy refers to decision-making, strategy, planning, and actions that aim to accomplish specific objectives and outcomes in the context of public health. Health regulation is a complex set of laws, rules, regulations, and procedures that set and update standards and ensure monitoring and compliance in health care.

Health policy and health regulation are closely related and may overlap. Their scope and scale may apply to local, regional, national, or even global populations. For example, during the COVID-19 pandemic, they formed a continuum of public health measures, rules, and laws that varied from one region to another and from country to country.

An array of organizations at different levels of government may be involved in the oversight and control of health policy and health regulation. Numerous private entities and commercial concerns may also provide input and influence outcomes. Therefore, there are often differences in perspective and tension between opposing interests. All these factors make health policy and health regulation more complex than clinical health services. These 2 areas of health care can be viewed as “complex health services.” Health care, then, can be broadly divided into clinical health services and complex health services. The latter encompasses health policy and health regulation and excludes clinical health services. Health care, as a whole, is transforming rapidly. In clinical health services, the advent of artificial intelligence (AI) and its real-world applications has resulted in a sea change. AI is now deployed in a raft of clinical health services, from medical imaging [1] to augmented reality microscopes [2] and from patient engagement to accurate diagnosis and treatment protocols.

AI algorithms are already better than human radiologists in identifying malignant tumors. AI-based smartphone apps offer an array of personalized services that support fitness, healthy lifestyles, health monitoring, and diagnosis. While AI has made important inroads across the entire spectrum of clinical health services, this is not the case, as yet, in complex health services. However, there is a rapid increase in the use of machine learning systems and sophisticated decision support in complex health services [3]. Humans still dominate this area, but AI is making quantum leaps in maturity, utility, and influence. It is only a matter of time before AI begins to drive, or dominate, complex health services as well. This may diminish the relevance of human decision makers in key areas of health policy and health regulation in the foreseeable future.

On the other hand, it is possible that humans may have certain unique attributes that influence decision-making, in this context, when compared to AI. For example, humans may offer a holistic and intuitive approach to decision-making [4] that may well present a competitive advantage to humans in future. Humans also have attributes that are a competitive disadvantage, such as escalation of commitment and sunk cost fallacy [5-7]. These attributes influence individuals or groups to persist in committing time, effort, and money to an outcome, even when that outcome has negative consequences.

Several theories seek to explain the basis of human decision-making. Expected Utility Theory [8] is a prominent theory in the field of economics that has been applied to health services. According to this theory, decision makers choose between possibilities that each carry a degree of risk, by comparing the expected utility of the possible choices. Expected Utility Theory is rooted in rationality and has given rise to 2 key concepts—cost-effectiveness and cost-utility. Cost-effectiveness focuses on the cost per unit of health improvement, while cost-utility evaluates the additional cost of a new treatment or intervention per unit of health improvement [9]. Cost-effectiveness and cost-utility can clash with the preferences of individual clinicians and patients [10], diminish equity in health care, and detract from the fair and objective allocation of resources [11]. Despite this, they underpin assumptions inherent in many modern health service models. For example, many models assume that cost-effectiveness influences decision-making to improve health care for a given population, even though it does not describe the value of the health improvement to the patient [9].

Numerous theories have sought to modify or challenge Expected Utility Theory. Bounded rationality [12] is one of the important modifications. Under bounded rationality, decision makers have limits, such as computational capacity, knowledge, organization, and memory usage. Prospect Theory [13] challenges Expected Utility Theory. It explores decision-making in the face of uncertainty and how people make decisions based on gain versus loss framing. This theory was particularly relevant in the COVID-19 pandemic, in an environment fraught with risk and highly emotional responses [14]. There is mounting evidence that decision-making may not be based on rationality alone [15]. Human beings are capable of making decisions using both intuition and reasoning [16-19]. Emotion also plays a major role in decision-making [20]. Researchers have sought to describe, distinguish, and differentiate cognitive processes based on rationality, on the one hand, and other ways of human decision-making, on the other [16,21,22]. These 2 cognitive processes can be viewed as System 1 and System 2 [22-24], which form the basis of Dual Process Theory.

Humans have the ability to apply some attributes internally and externally, such as behavioral flexibility [25] and cognitive complexity [26]. Competencies such as advanced adaptive expertise [27], dialectical thinking [28], and neuroplasticity [29] allow humans to make nuanced decisions. In contrast, attributes such as cognitive bias [23,30-32] may lead to an overreliance on previous knowledge or expected observations, which can result in suboptimal decisions. However, cognitive bias may improve the efficiency of decision-making when used in combination with heuristics [33]. Heuristics are rough, rule-of-thumb guides that reduce the effort needed to make decisions—mental strategies that allow decisions to be made easily and quickly [33]. The availability heuristic, representative heuristic, and anchoring and adjustment heuristic can enhance complex decision-making. When combined with other factors that influence decision-making, such heuristics form an important part of critical thinking [32]. However, heuristics can result in errors and bias—for example, the representative heuristic can propagate stereotypes [34].

Decision-making in complex health services needs to address the uncertainty of foreseeable events. It also needs to consider and address the radical uncertainty of unimaginable events [35]. Radical uncertainty refers to events such as the COVID-19 pandemic, where decisions and actions lead to outcomes that were profoundly uncertain. In such situations, it is challenging or impossible to establish the structure of the problem at hand, determine probabilities based on a comprehensive list of knowable outcomes, or choose among various possibilities [36-39]. In the current era, which is dynamic, connected, and complex, important decisions are made under radical uncertainty across many domains, including economics, finance, politics, and government [40]. Conviction Narrative Theory (CNT) is a framework for decision-making under radical uncertainty [40]. CNT proposes that in radical uncertainty, decision makers should build narratives that map the future outcomes of all proposed actions. They should then develop enough conviction to make a decision by selecting an action. In complex health services, CNT is relevant in contexts such as the COVID-19 pandemic, which required decisions to be made at speed.

### Rationale and Objectives

Many of the assumptions inherent in health service models today are underpinned by Expected Utility Theory [8]. For example, cost-effectiveness is a rational measure that is often considered one of the most important criteria for decisions on health care improvements for a given population [9]. Such rational, evidence-based metrics currently dominate the culture of decision-making in health policy and regulation. However, as the COVID-19 pandemic has shown, there are other important considerations in these complex spheres of health care, such as ethical and moral considerations. Rational metrics such as data, statistics, and cost alone may not suffice in making better decisions in these health care domains. Identifying and analyzing attributes that influence decision-making, not only within the bounds of rationality but also beyond it, may have ramifications for decision-making in these important spheres of health care. Therefore, this scoping review was undertaken to identify and map the attributes that influence human decision-making in complex health services that have been reported in the peer-reviewed literature.

### Review Question

This scoping review was designed to answer the following research question:

- What attributes have been reported in the literature that influence human decision-making in complex health services?

### Framework

This scoping review is reported in accordance with the framework and recommendations by Peters et al [41]. The population of interest consists of human decision makers. The concept is decision-making in the context of complex health services. As the topic of interest merited broad review to scope and understand literature from a holistic viewpoint, a scoping review of literature was appropriate here.

## Methods

### Study Design

This scoping review provides a clear, reproducible methodology [42] and conforms to the reporting guidelines presented in the PRISMA-ScR (Preferred Reporting Items for Systematic Reviews and Meta-Analyses Extension for Scoping Reviews) [43].

### Search Strategy

All available databases were included within each of 4 search systems—ProQuest, Scopus, PubMed, and Web of Science. Search terms and a search strategy were defined for each of these systems (Multimedia Appendix 1). The most recent search was undertaken on June 9, 2023. Once the search results were evaluated and relevant papers identified, manual citation tracking was also undertaken—a snowball search of all the references within the papers deemed relevant.

### Inclusion and Exclusion Criteria

All selected search systems contain papers from 1976 onward. Therefore, this was selected as the “start” year of publication. To include recent research, 2022 was the “end” year selected. Only papers in English where included, in the interest of time—papers in other languages were excluded. All papers relating to human decision-making in complex health services were included. Papers that focus on topics not relevant to the research question were excluded. Multimedia Appendix 2 lists the inclusion and exclusion criteria applied. The most recent search was conducted on June 9, 2023.

### Data Extraction

The first author removed duplicates from the database search results and read the titles and abstracts of the remaining papers—or, where abstracts were not available, the full text of the papers. The first author then read the full text of the remaining papers, applying inclusion and exclusion criteria until only relevant papers remained. The second author reviewed this. The extracted data was cross-checked by both authors to minimize personal bias [44]. Any disagreements on data extraction and the categorization of papers were resolved through detailed discussions, leading to consensus between the authors.

### Data Analysis

A thematic analysis was undertaken in order to identify the human attributes mentioned in the literature reviewed, enable a frequency count of attributes, and map these results in diagrammatic or tabular form.

## Results

The results span 46 years, from 1976 to 2022. Overall, 167 papers were identified, and 86 duplicates removed. The titles and abstracts of the remaining 81 papers were screened, based on inclusion and exclusion criteria. This process resulted in the exclusion of 69 papers. Both authors read the full text of the remaining 12 papers. Of these, 8 were excluded because they neither related to complex health services nor specifically mentioned health policy or health regulation. The remaining 4 papers were found to be relevant to the research question.

**Figure 1:**
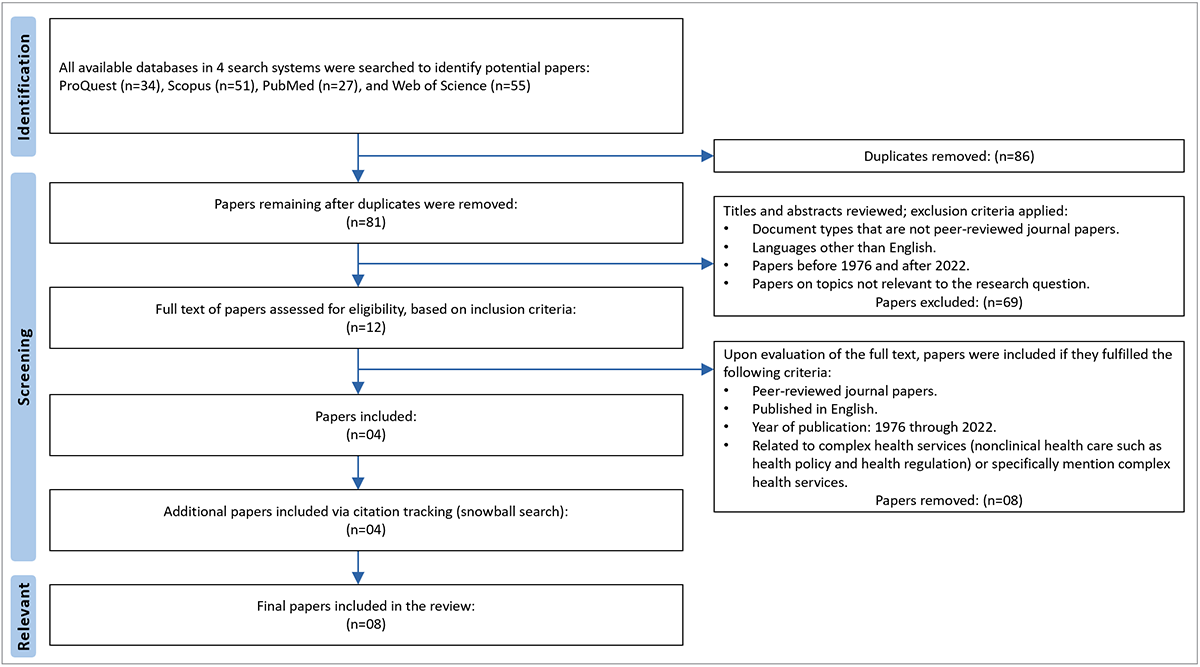
Flow diagram based on the PRISMA-ScR (Preferred Reporting Items for Systematic Reviews and Meta-Analyses Extension for Scoping Reviews).

Citation tracking was then undertaken—a snowball search of all references within these 4 papers. This process identified 4 more relevant papers. Thus, a total of 8 relevant papers were included. Figure 1 [43] shows the PRISMA (Preferred Reporting Items for Systematic Reviews and Meta-Analyses) flow diagram of paper screening and selection. A PRISMA-ScR checklist is also included in Multimedia Appendix 3.

The key results relevant to the research question are presented below.

**Figure 2:**
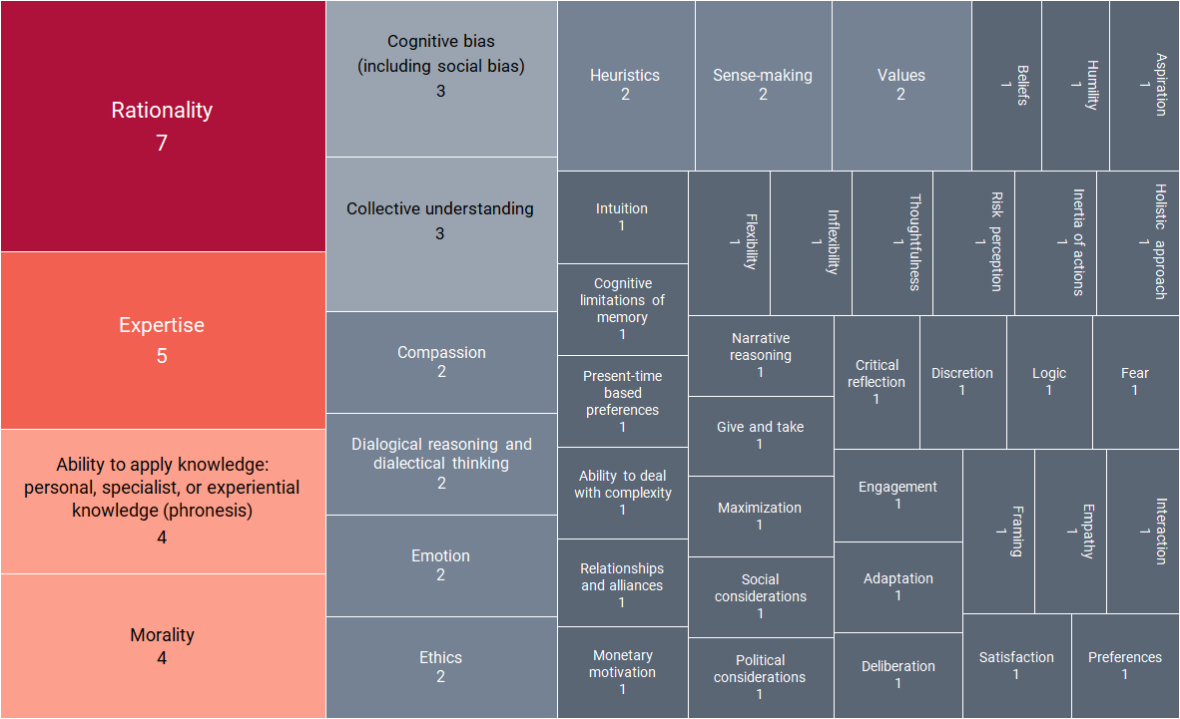
Human attributes (n=45) that influence decision-making in complex health services: frequency of mentions in included papers.

- The included papers were reviewed in detail to identify the human attributes mentioned and count the frequency of mentions (Figure 2).
  A total of 45 human attributes were identified.
  Rationality is mentioned in 7 of the 8 papers—it is the most frequent attribute mentioned.
  This is followed by expertise, mentioned in 5 papers.
  Morality is mentioned in 4 papers.
  The ability to apply personal, specialist, or experiential knowledge (phronesis) is mentioned in 4 papers.
- Two key themes were identified (Multimedia Appendix 4 [45-52]).
  The complexity of human decision-making in complex health services, various aspects of which are discussed in 6 of the papers.
  Cognitive processes involved in decision-making in complex health services, which are discussed in 2 of the papers included.

## Discussion

### Principal Findings

The selected papers lend credence to the hypothesis that rationality alone may not suffice in making better decisions in complex health services. Carminati [45] postulates that humans tend to make decisions that are not always rational. Humans also have a limited capacity for information processing, relying on heuristics to make judgements and decisions. In the health care sector, decisions are based on information that is limited and asymmetrical, despite the critical and urgent choices that often need to be made. Therefore, it may be useful to apply perspectives from behavioral economics because it is based on social sciences such as sociology and psychology.

Lechanoine and Gangi [46] state that cognitive biases such as the belief bias and availability bias often challenge our rational thinking. Humans also rely on heuristics to process information that enables them to arrive at judgments and choices. A reliance on the representativeness heuristic, for instance, may result in overestimating the likelihood of low-risk events occurring and underestimate high-probability risks. Humans also use the bandwagon effect, doing things because others are doing them.

Gaissmaier [47] argues that understanding attributes such as risk perception may require a cognitive-ecological lens that assesses interactions between cognitive processes and the environment. Russell and Greenhalgh [48] postulate that being “human” is not the antithesis of being “rational”—instead, both are important to making better decisions. Emotions bring power and value in clarifying what is important to human beings, in the context of decision-making in complex health care. Furthermore, in these types of decisions, there is value in using embodied rationality [48], which recognizes the body, emotions, and the “irrational” unconscious [53].

Greenhalgh and Russell [49] argue that a purely rational, evidence-based framework for health policy decisions does not allow the proper consideration of complex, competing options, because these options are often values-based and dependent on context. These authors suggest that the sociolinguistic mechanisms of argumentation theory, negotiation, collective deliberation, and “muddling through,” may enhance the quality and richness of decisions made in complex health care, particularly in the face of competing values and under conditions of uncertainty.

In the context of health policy decisions, O’Brien-Pallas and Baumann [50] state that evidence-based facts and research findings alone may not be sufficient to make the best decision or determine the optimal course of action. Tenbensel [51] argues that prioritizing rational considerations such as cost-utility may not result in effective health policy, because it devalues specialist expertise and lay experience. Mechanic [52] states that it is clinical experience and nuanced judgement, more than science and rationality, that influence decisions that impact a patient’s lived experience and response to care. However, at the policy level, bureaucrats often do not take these complex factors into account, and develop explicit policies and standards based solely on rationality instead.

In the papers included, 45 attributes were identified (Figure 2). Rationality is the most frequently mentioned human attribute (n=7). Other attributes based on rationality are also mentioned frequently—for instance, expertise (n=5), and the ability to apply knowledge (n=4). However, the findings also reflect a wider acceptance and acknowledgment that human decision-making is based on more than just rationality and the attributes associated with it. Morality is mentioned 4 times, cognitive bias and collective understanding receive 3 mentions each, with attributes such as dialogical thinking and emotion receiving 2 mentions each.

The methods used in this scoping review are as rigorous and transparent as possible. The framework described by Peters et al [41] was adopted as a useful, contemporary guide. An informal exploration was undertaken to determine optimal electronic search systems. This resulted in the selection of 4 search systems that contain many subject areas relevant to the research question. The search strategy included a database search of all databases available in these systems, as well as citation tracking.

This scoping review has limitations. Searching other systems and bibliographic databases may have yielded additional results. This review only includes peer-reviewed journal papers published in English and papers published from 1976 to 2022. These limiters may well have resulted in missing some relevant papers.

### Conclusion

The objective of this scoping review was to identify and map the attributes that influence human decision-making in complex health services that have been reported in the peer-reviewed literature. A total of 45 attributes were identified and mapped according to the frequency of mentions. Rationality was the most frequently mentioned attribute, followed by other attributes based on rationality, such as expertise and the ability to apply knowledge. The results indicate that rationality is entrenched and may influence the lexicon of our thinking about decision-making. However, the findings also highlight other attributes such as morality, cognitive bias, and collective understanding, which may be considered more intuitive than rational. The results highlight the counter narrative of decision-making underpinned by uniquely human attributes.

In total, 2 key themes emerge from an analysis of the papers included in this review—the complexity of human decision-making and the cognitive processes involved in decision-making. These themes underline the complex and nuanced nature of human decision-making, which involves many cognitive processes based not only on rationality but on emotions as well. Therefore, this scoping review may have real-world, practical value, with ramifications for decision-making in complex health services today. The review itself has taken a rational approach, and the methods used were suited to this. However, there may be scope to take a more intuitive approach.

## Appendix

Multimedia Appendix 1 Search systems, databases, and search terms used to identify literature for review. Search conducted on 9 June, 2023.

Multimedia Appendix 2 Inclusion and exclusion criteria.

Multimedia Appendix 3 Preferred Reporting Items for Systematic Reviews and Meta-Analyses Extension for Scoping Reviews (PRISMA-ScR) checklist.

Multimedia Appendix 4 Key features and categorization of papers included, listed in chronological order.

## Abbreviations

AI: artificial intelligence
CNT: Conviction Narrative Theory
PRISMA: Preferred Reporting Items for Systematic Reviews and Meta-Analyses
PRISMA-ScR: Preferred Reporting Items for Systematic Reviews and Meta-Analyses Extension for Scoping Reviews

## Data Availability

All data generated or analyzed during this study are included in this published paper and its supplementary information files.
