# Supplementary material for: Attributes That Influence Human Decision-Making in Complex Health Services: Scoping Review
Source: JMIR Hum Factors. 2023 Dec 20;10:e46490. doi: 10.2196/46490 (PMC10765291; doi:10.2196/46490)
Supplement: Multimedia Appendix 1 [file humanfactors_v10i1e46490_app1.pdf]

Multimedia Appendix 1

Search Systems, Databases, and Search Terms Used to Identify Literature for Review. Search Conducted on 9 June 2023.

Search System / Databases	Search Terms	Number of Papers
ProQuest<br>(All ProQuest databases)<br><br><br><br><br>Limiters:	TI(decision) AND TI(human) AND TI(health) NOT papillomavirus NOT AB(clinical) NOT virus NOT Fukushima<br>Expansion of abbreviations:<br>TI=Title<br>AB=Abstract<br><br>Type of paper: Peer-reviewed journal papers.<br>Languages: English only.<br>Year of publication: 1976 to 2022.	34
Scopus<br>(The Scopus database)<br><br><br><br>Limiters:	TITLE(decision) AND TITLE(human) AND TITLE(health) AND NOT papillomavirus AND NOT ABS(clinical) AND NOT virus AND NOT Fukushima<br>Expansion of abbreviations:<br>ABS=AbstractAbstract<br><br>Type of paper: Peer-reviewed journal papers.<br>Languages: English only.<br>Year of publication: 1976 to 2022.	51
PubMed<br>(Primary focus: Life sciences and biomedical topics)<br><br>Limiters:	((((((decision[Title]) AND (human[Title])) AND (health[Title])) NOT (papillomavirus)) NOT (clinical[Title/Abstract])) NOT (virus)) NOT (Fukushima) Abstract<br><br>Type of paper: Peer-reviewed journal papers.<br>Languages: English only.<br>Year of publication: 1976 to 2022.	27

Search System / Databases	Search Terms	Number of Papers
Web of Science<br>(All Web of Science databases)	TI=(decision AND human AND health) NOT TS=(papillomavirus) NOT AB=(clinical) NOT TS=(virus) NOT TS=(Fukushima)<br>Expansion of abbreviations:<br>TI=Title<br>AB=Abstract<br>TS=Topic	55
Limiters:	Type of paper: Peer-reviewed journal papers.<br>Languages: English only.<br>Year of publication: 1976 to 2022.	
Total records identified by the database search		167
Total records after duplicates were removed		81
