# Supplementary material for: Attributes That Influence Human Decision-Making in Complex Health Services: Scoping Review
Source: JMIR Hum Factors. 2023 Dec 20;10:e46490. doi: 10.2196/46490 (PMC10765291; doi:10.2196/46490)
Supplement: Multimedia Appendix 2 [file humanfactors_v10i1e46490_app2.pdf]

Multimedia Appendix 2

Inclusion and Exclusion Criteria

Inclusion criteria	Exclusion criteria
Paper type	
Peer-reviewed journal papers	All other paper types
Language	
English	All other languages
Year of publication	
1976 - 2022	Papers published before 1976 and after 2022
Other criteria	
Papers were included if they:<br>• related to complex health services (nonclinical health care such as health policy and health regulation); or<br>• specifically mentioned complex health services such as health policy and health regulation.	Papers on topics not relevant to the research question were excluded – topics such as:<br>• clinical health;<br>• health workforce;<br>• legal matters;<br>• environmental health, contamination, and toxicity;<br>• computers, human-computer interaction, and automated decision rules;<br>• mathematical modelling; and<br>• assessment of organisational performance.
