# Supplementary material for: Attributes That Influence Human Decision-Making in Complex Health Services: Scoping Review
Source: JMIR Hum Factors. 2023 Dec 20;10:e46490. doi: 10.2196/46490 (PMC10765291; doi:10.2196/46490)
Supplement: Multimedia Appendix 4 [file humanfactors_v10i1e46490_app4.pdf]

Multimedia Appendix 4

Key Features and Categorization of Papers Included, Listed in Chronological Order

Paper No.	Author and Year of Publication	Title	Source and Type of Paper	Relevant data that relates to the review question and objective	Key Theme	Attributes Mentioned in the Paper
1	Carminati, L [45]<br>2020	Behavioral economics and human decision making: Instances from the health care system	ProQuest<br>Review of literature	Humans tend to make decisions that are not always rational. Humans also have a limited capacity for information processing, relying on heuristics to make judgements and decisions. In the health care sector, decisions are based on information that is limited and asymmetrical, despite the critical and urgent choices that often need to be made. Therefore, it may be useful to apply perspectives from behavioural economics because it is based on	Complexity of human decision making in complex health services	• Rationality<br>• Cognitive limitations of memory and ability to acquire information<br>• Heuristics<br>• Present-time based preferences<br>• Satisfaction versus maximisation<br>• Monetary motivation<br>• Inertia of actions<br>• Framing<br>• Collective understanding (considering shared social norms)

Paper No.	Author and Year of Publication	Title	Source and Type of Paper	Relevant data that relates to the review question and objective	Key Theme	Attributes Mentioned in the Paper
				social sciences such as sociology and psychology.		
2	Lechanoine, F, Gangi, K [46]<br>2020	COVID-19: Pandemic of cognitive biases impacting human behaviors and decision making of public health policies	Web of Science<br>Opinion	Cognitive biases such as the belief bias and availability bias often challenge our rational thinking. Humans also rely on heuristics to process information that enables them to arrive at judgments and choices. A reliance on the representativeness heuristic, for instance, may result in overestimating the likelihood of low-risk events occurring and underestimate high-probability risks. Humans also use the bandwagon effect, doing things because others are doing them.	Cognitive processes in formulating health policy	• Rationality<br>• Cognitive bias<br>• Heuristics

Paper No.	Author and Year of Publication	Title	Source and Type of Paper	Relevant data that relates to the review question and objective	Key Theme	Attributes Mentioned in the Paper
3	Gaissmaier, W [47]<br>2019	A cognitive-ecological perspective on risk perception and medical decision making	Citation tracking / snowball search<br>Commentary	Understanding attributes such as risk perception may require a cognitive-ecological lens that assesses interactions between cognitive processes and the environment.	Cognitive processes in formulating health policy	• Fear<br>• Cognitive bias<br>• Risk perception
4	Russell, J, Greenhalgh, T [48]<br>2014	Being 'rational' and being 'human': How National Health Service rationing decisions are constructed as rational by resource allocation panels	Web of Science<br>Rhetorical analysis	Being 'human' is not the antithesis of being 'rational' - instead, both are important to making better decisions. Emotions bring power and value in clarifying what is important to human beings, in the context of decision making in complex health care. Furthermore, in these types of decisions, there is value in using embodied rationality, which recognises the body,	Complexity of human decision making in complex health services	• Rationality<br>• Dialogical reasoning<br>• Values<br>• Ethics<br>• Morality<br>• Emotions<br>• Narrative reasoning<br>• Sense making<br>• Intuition<br>• Holistic approach<br>• Empathy<br>• Critical reflection<br>• Ability to use experiential

Paper No.	Author and Year of Publication	Title	Source and Type of Paper	Relevant data that relates to the review question and objective	Key Theme	Attributes Mentioned in the Paper
				emotions, and the 'irrational' unconscious.		knowledge (phronesis)
5	Greenhalgh, T, Russell, J, [49]<br>2009	Evidence-based policymaking: A critique.	Citation tracking / snowball search<br>Critique	A purely rational, evidence-based framework for health policy decisions does not allow the proper consideration of complex, competing options, because these options are often values-based and dependent on context. Sociolinguistic mechanisms of argumentation theory, negotiation, collective deliberation, and 'muddling through', may enhance the quality and richness of decisions made in complex health care, particularly in the face of competing	Complexity of human decision making in complex health services	• Rationality<br>• Logic<br>• Collective understanding<br>• Dialectical thinking<br>• Ability to apply personal knowledge<br>• Ethics<br>• Morality<br>• Interaction<br>• Adaptation<br>• Sense-making<br>• Deliberation

Paper No.	Author and Year of Publication	Title	Source and Type of Paper	Relevant data that relates to the review question and objective	Key Theme	Attributes Mentioned in the Paper
				values and under conditions of uncertainty.		
6	O'Brien-Pallas, L, Baumann, A [50]<br>2008	Toward evidence-based policy decisions: A case study of nursing health human resources in Ontario, Canada	ProQuest<br>Case study	In the context of health policy decisions, evidence-based facts and research findings alone may not be sufficient to make the best decision or determine the optimal course of action.	Complexity of human decision making in complex health services	• Rationality<br>• Values<br>• Beliefs<br>• Collective understanding
7	Tenbensel, T [51]<br>2000	Health prioritisation as rationalist policy making: Problems, prognoses and prospects	Citation tracking / snowball search<br>Commentary	Prioritising rational considerations such as cost-utility may not result in effective health policy, because it devalues specialist expertise and lay experience.	Complexity of human decision making in complex health services	• Rationality<br>• Expertise<br>• Specialist knowledge<br>• Generic expertise<br>• Technical expertise<br>• Medical expertise<br>• Relationships and alliances<br>• Morality and moral considerations<br>• Compassion

Paper No.	Author and Year of Publication	Title	Source and Type of Paper	Relevant data that relates to the review question and objective	Key Theme	Attributes Mentioned in the Paper
8	Mechanic, D [52]<br><br>1997	Muddling through elegantly: Finding the proper balance in rationing	Citation tracking / snowball search<br><br>Commentary	Clinical experience and nuanced judgement, more than science and rationality, influence decisions that impact a patient's lived experience and response to care. However, at the policy level, bureaucrats often do not take these complex factors into account, developing explicit policies and standards based solely on rationality instead.	Complexity of human decision making in complex health services	• Rationality<br>• Inflexibility and flexibility<br>• Social considerations<br>• Moral considerations<br>• Political considerations<br>• Thoughtfulness<br>• Medical expertise<br>• 'Give and take'<br>• Compassion<br>• Discretion<br>• Emotion<br>• Aspiration<br>• Preferences<br>• Social bias<br>• Ability to deal with complexity<br>• Engagement<br>• Humility
